# Operationalising One Health in Nigeria: Reflections From a High-Level Expert Panel Discussion Commemorating the 2020 World Antibiotics Awareness Week

**DOI:** 10.3389/fpubh.2021.673504

**Published:** 2021-05-31

**Authors:** Chioma R. Achi, Olaniyi Ayobami, Godwin Mark, Abiodun Egwuenu, David Ogbolu, Junaidu Kabir

**Affiliations:** ^1^Department of Veterinary Medicine, University of Cambridge, Cambridge, United Kingdom; ^2^Department of Veterinary Public Health and Preventive Medicine, Usmanu Danfodiyo University, Sokoto, Nigeria; ^3^Department of Infectious Disease Epidemiology, Robert Koch Institute, Berlin, Germany; ^4^Department of Internal Medicine, Federal Teaching Hospital Gombe, Gombe, Nigeria; ^5^National AMR Programme, Nigeria Centre for Disease Control, Abuja, Nigeria; ^6^Medical Microbiology Unit, Department of Medical Science, Ladoke Akintola University of Technology, Ogbomosho, Nigeria; ^7^Department of Veterinary Public Health and Preventive Medicine, Ahmadu Bello University Zaria, Zaria, Nigeria

**Keywords:** one health, antimicrobial stewardship, antimicrobial resistance, sub-Sahara Africa, rational usage, health system

## Abstract

Antimicrobial resistance (AMR) poses an important One Health challenge for all countries of the world. As human, animal and environmental health are closely linked, it is essential that interventions targeted at reducing the spread of AMR and those promoting antimicrobial stewardship are conducted with all sectors in mind. Tackling this global slow-moving pandemic (AMR) also requires action and strong commitment from all countries of the world. Nigeria, like many other countries, have made considerable progress in implementing the National Action Plan on Antimicrobial Resistance. The accomplishments and ongoing work led by the National Technical Working Group on AMR is commendable. However, gaps still exist in terms of operationalising One Health interventions for AMR, especially regarding rational antimicrobial use and antimicrobial stewardship. The 2020 World Antimicrobial Awareness Week presented an opportunity to convene a multi-sectoral expert panel from national government agencies, research, academia and the World Health Organisation across the Nigerian One Health space. The panel discussion analysed the progress made so far and identified the barriers and the opportunities for operationalising One Health interventions on AMR. The discussion highlighted poor awareness and the *fear* phenomenon, driven by technical and socio-economic factors, as a common cross-sectoral denominator at the heart of inappropriate antibiotic use within the country. At the system level, suboptimal use of antimicrobials fuelled by the ease of purchase, poor regulations and insufficient enforcement of prescription-only access to antimicrobials, and limited infection prevention and biosecurity measures resonated as drivers of AMR across One Health sectors in Nigeria. Looking forward, the panel discussion identified substantial investment in the governance of the existing One Health component structures, inclusive bottom-up institutional antimicrobial stewardship that fosters community participation and multi-level cross-sectoral collaborations as the next level strategic imperatives. In this respect is the need for a strengthened One Health infrastructure, including an operational workforce, educational strategies to elevate AMR and rational antimicrobial use into public consciousness, and the use of improved data systems as countermeasures to the challenge of AMR.

## Background to the Challenge of Antimicrobial Resistance

Antimicrobial resistance (AMR) is the ability of microbes to survive exposure to an antimicrobial agent (through inherent mutation or gene acquisition) that had previously inhibited or killed it. AMR is a natural characteristic of microbes accelerated by anthropogenic factors such as inappropriate antibiotic use, global environmental changes, and travel, among others. Indeed, the overuse and misuse of antibiotics in humans and animals have been an established driver of AMR (1–3). The interconnection between animal, human and environmental health, and how infectious pathogens, as well as resistance genes, move among these interfaces is also well-established (1, 2, 4), making AMR an important One Health challenge of global concern.

The Jim O'Neil Report of 2014 considered AMR a global crisis with three sub-dimensions: a health crisis, an economic crisis, and a security crisis (5). AMR's direct impact heightens death risks from common infections, resulting in a significant health crisis. AMR make illnesses longer and more severe, prolong hospital stays and escalates healthcare costs. There is also an increased rate of therapeutic failure leading to loss of protection for patients undergoing common surgeries like caesarean sections and hip replacements; as one in six routine procedures will end in death if antibiotics lose their effectiveness (6, 7). As an economic crisis, the 2014 World Health Organisation (WHO) estimate suggests that the cost to society for AMR is over US$35 billion each year (8). Up to 700,000 deaths, including 214,000 neonatal sepsis deaths are estimated to result from resistant bacterial pathogens each year (as of 2016), with projections predicting 10 million deaths by 2050 if there is no urgent intervention to halt AMR sustainably (5). Even though inadequate access and delays in access to antibiotics cause more deaths than antibiotic resistance, more deaths associated with AMR are being reported in many countries regardless of the level of development.

## AMR Situation in Nigeria

Nigeria considers AMR a priority on the national public health agenda. The Nigeria response to antimicrobial resistance led by the Nigeria Centre for Disease Control (NCDC) began in 2016 following a situational analysis that investigated common antimicrobial-resistant pathogens recovered from hospitals, animal sources, agricultural and environmental sources. The NCDC also conducted systematic reviews to assess the prescribing patterns of antimicrobials in hospitals across the country. These efforts informed the development of the National Action Plan (NAP), designed with a 5-year focus in mind (2017–2022). Overall, the Nigeria NAP seeks to address five key pillars in consonance with the WHO Global Action Plan on AMR. The pillars include increasing awareness and knowledge of health workers and the general public on AMR; building a One Health surveillance system; intensifying infection prevention and control and biosecurity; promoting rational use of antimicrobials and access to quality medications, and research into alternatives to antimicrobials, new diagnostics and therapeutics.

In this light, a multi-sectoral expert panel - drawn across national government agencies, research, academia and the World Health Organization (WHO)- active in the One Health space was convened. The panel includes program managers, researchers, clinicians and Public Health practitioners from the Federal Ministries of Health, Agriculture and Environment, national universities, teaching hospitals and international partners like the WHO. The panel discussion focused on identifying opportunities, barriers to operationalising one health approaches in the management of antimicrobials to understand and manage the AMR crisis with a contextual appreciation of developments in Nigeria.

One of these strategic interventions identified for a One-Health action in Nigeria is the promotion of rational use of antibiotics and access to quality medications (9, 10). The Technical Working Group (TWG) on AMR [The Antimicrobial Resistance Coordinating Committee (AMRCC)] led by the NCDC is a multi-sectoral team drawn from animal, environmental and human health sectors. So far, with support of the international partners, it has equipped 11 laboratories in the human sector and seven in the animal sector, and alongside its partners and the TWG, continues to support these laboratories to upscale the surveillance of antimicrobial-resistant pathogens within the country. Other notable activities include antimicrobial stewardship interventions, point prevalence surveys to establish antimicrobial prescribing and antimicrobial use across hospitals, and to estimate the burden of healthcare-associated infections across participating facilities.

The Federal Ministry of Agriculture and Rural Development (FMARD) formulate policies that cover AMR in terrestrial and aquatic animals. It has a focal point for AMR and veterinary products that resides in the Federal Department of Veterinary and Pest Control services. FMARD, therefore, reports data on Antimicrobial use (AMU) to the World Organisation for Animal Health (OIE). The FMARD reported that over 300,000 kg of antimicrobial agents is imported into the country on an annual basis for use within the animal health sector some of which are in the list of critical antimicrobials under the OIE classification. In recent time, the support of international partners has enabled AMR surveillance activities in the animal health sector and their engagement with the World Health Organisation (WHO) Tricycle project on AMR and AMU.

The environmental health sector reports its active implementation of awareness programs on AMR mostly linked to the roles that hygiene, sanitation and proper waste disposal play in curtailing the spread of pathogens, antimicrobial-resistant bugs inclusive. However, there are still notable gaps in monitoring AMR in effluents and undertaking the much-needed surveillance programs within the sector due to myriads of factors, including limited funds. Currently, there are no clearly defined roles for the major players in the ministry of environment regarding surveillance and control of AMR pathogens.

## Antimicrobial Use and the Sectoral Drivers of Antimicrobial Resistance in Nigeria

Antimicrobials are at the centre of an exploding interaction that drives the emergence of AMR, which in turn undermines human health, animal health, agriculture, and environmental sustainability. The ease with which antimicrobials are accessed and bought over the counter flourishes under a climate of a poorly regulated antimicrobial market and the inadequate enforcement of prescription-only-access to antibiotics where required. In many cases, over the counter access is not limited to first or second-line antibiotics alone but cuts across even the critically important class of antimicrobials, some of which are “hawked” on the street in some regions of the country. The discussion highlighted physicians' experiences with limited options to handle life-threatening cases, often as a result of patients exhausting all classes of antimicrobials, including third-generation cephalosporins, available to them from patent medicine stores before visiting the hospital. There is also the problem of improper disposal of expired antibiotics and left-over medicines by the general public, which mostly end up in conventional waste bins and dumps due to lack of awareness on the part of the users and weak enforcement on the part from regulatory authorities.

The challenge of indiscriminate use cuts across human health and animal sectors. In the human health sector, most cases of fever are treated empirically with antimicrobials. Although some of the antimicrobials administered are prescriber-led, patients and their relatives often pressurise healthcare workers for antimicrobials, and in most cases, venture into self-medication with antimicrobials even for simple viral infections.

The need to prevent the occurrence of diseases or infections within flocks, herds or aggregations, on the one hand, is a major reason for the high use of antimicrobials within the animal health sector. Many farmers routinely incorporate minute doses of antimicrobials into the animal feed or water to enhance growth and, in the case of poultry production, to increase egg size in order to guarantee more income from sales. Feed manufacturers also incorporate antimicrobials to improve the animals' feed efficiency, thus maintaining patronage from farmers. Antimicrobial stewardship appears to be low among farmers, as some do not understand the need to observe the withdrawal periods of medicines before the slaughter of the animals, or where they do, sometimes fail to observe them. Before now, the lack of specific regulations regarding AMU in the animal health sector provided a wide window for farmers and non-professionals to freely purchase antimicrobials from the many veterinary pharmacies and stores within the country. However, the recently revised Essential Veterinary Drug List document of the Nigeria Veterinary Council has a provision on classification and guide to use of antimicrobial agents clearly specifying which drugs are over-the-counter drugs.

Antimicrobials in their spray form are frequently used to wade off pests and keep off pathogens in crop production. Anecdotally, some farmers within the country also incorporate antimicrobials, pesticides and sometimes some organophosphates during the storage of grains to preserve the harvest for long-term gains.

Consequently, human and animal waste products, particles from sprays used on crops, discharge from hospitals, pharmaceutical facilities and farms, carrying antimicrobial-resistant pathogens and remnants of antimicrobials, find their way into the environment. This creates a vicious cycle that adversely affects crops production, water bodies as well as aquaculture.

### The Fear Phenomenon as a Cross-Sectoral Social Driver of Antimicrobial Use and Resistance

**Figure 1:**
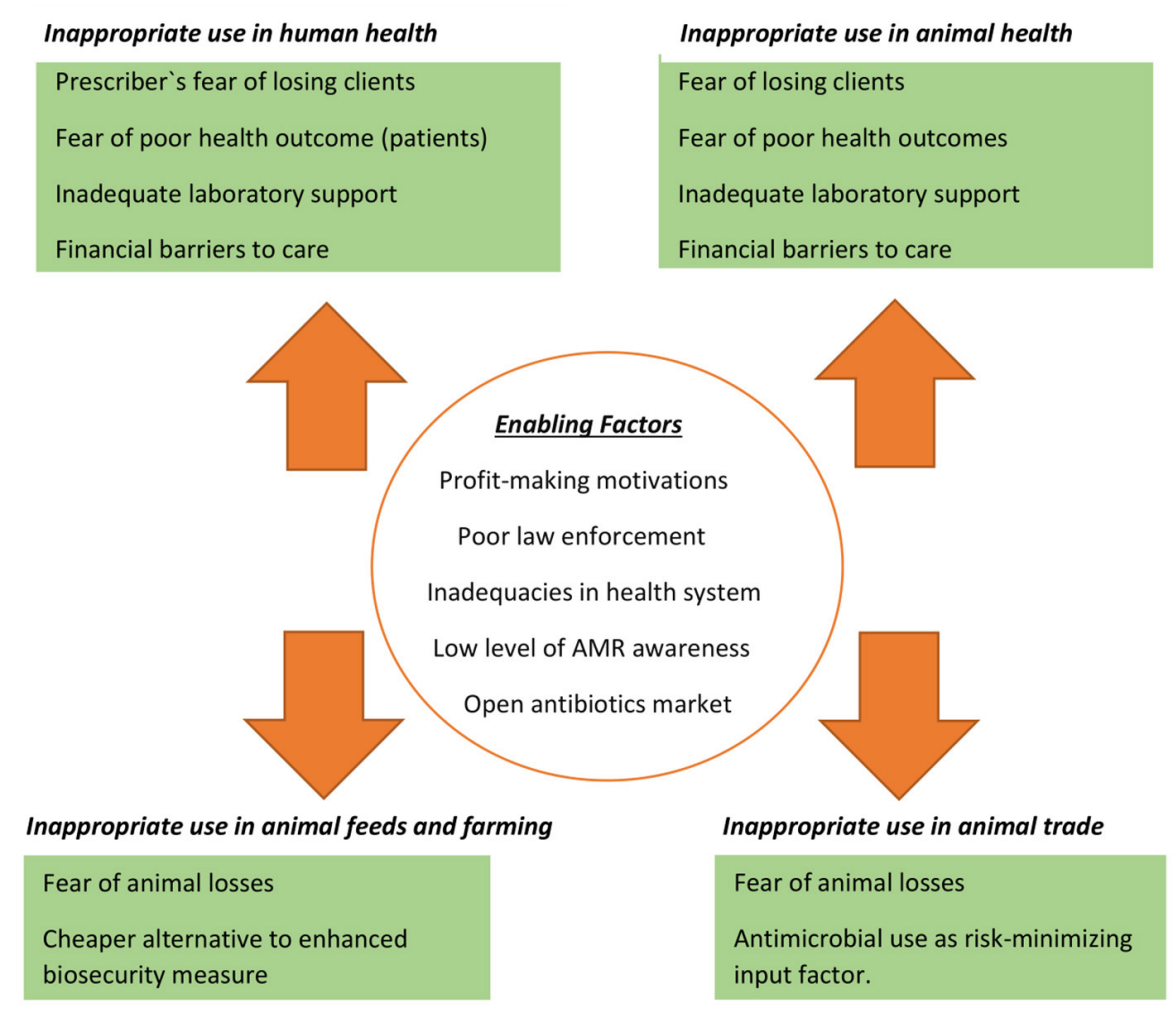
Fear as an underlying motivation for cross-sectoral suboptimal antibiotic use and its enabling factors.

On many fronts, the place of fear as a driver of AMR cannot be over-emphasised (Figure 1). Many patients fear that their infections might worsen if they do not address them by immediately taking antimicrobials. This is exacerbated by the challenge of waiting long hours on hospital queues only to spend more money running laboratory tests or to pay for consultations that might cost the same amount or even more when compared to the cost of a full dose of a common antimicrobial. As physicians and healthcare workers' capability are usually judged by the patients' outcome following an illness, many healthcare workers fear that their reputation might be at stake if their patients do not do well. Hence, the temptation to challenge all pathogens at the same time with an “antimicrobial-cover.” Many privately-owned hospitals and clinics often depend on “patient advertising” to increase their client base, and they fear that their patients might leave them for another provider if they do not give in to the pressure of prescribing antimicrobials. Therefore, it is a common finding in private practise for physicians to give in to patient pressure and demand for antimicrobials to increase the hospital income.

The fear factor also features prominently in the agriculture and animal health sector. Large- or small-scale farming is a source of livelihood and a worthy economic venture for retirees making farmers more likely to do all within their power to secure their investments; in many cases, administering a cocktail of antimicrobials daily or weekly. Simply put, the fear of losing their animals and their investments is one major factor that drives AMU, hence AMR. In animal markets, it is not uncommon for traders as a way of recouping cost to administer antimicrobials to their animals while awaiting buyers. They do this out of fear of losing their animals before sale, and for fear of a diseased animal spreading an infection to other animals meant for sale. In addition to farmers and traders administering antimicrobials out of fear, veterinarians and other animal health workers also administer and prescribe antimicrobials as a magic bullet to get rid of any infection and for fear of losing clients that might question their competency and skills.

Some agricultural extension workers also encourage farmers to routinely use antimicrobials for wading off pathogens on plants and crops; they also do this as a way of improving plant health. The resultant effect of fear in terms of AMU is the propagation of AMR's transmission cycle, where pathogens that are regularly exposed to antimicrobials develop resistance to the panel of antimicrobials used over the years. This overuse drives selective pressure within the environment that enables the emergence and spread of resistant infections.

Fear is a common denominator in the Nigerian One Health space driven by technical and socio-economic factors; in the case of animal production, mostly driven by the absence of compensations and wavering support from the Government for losses incurred.

### Infection Prevention and Control, Diagnostic and Biosecurity Infrastructure Is Still Limited

Poor infrastructure limits the practice of infection prevention and control (IPC) in healthcare settings and biosecurity measures on farms. Constant water supply and basic hand-washing facilities are scarce resources for most healthcare facilities and farms within the country. It is challenging to prevent the spread of infections within and outside healthcare settings if the basic infrastructure to improve hygiene and sanitation is lacking. In the face of inadequate biosecurity measures, many farmers resort to prophylactic use of antimicrobials to prevent on-farm infections or outbreaks of diseases, increasing AMU and AMR.

Many healthcare facilities and centres do not have resources such as incinerators and consumables to properly treat healthcare wastes (sometimes hazardous and infectious) before disposal. The lack of essential resources for waste disposal means that all waste is treated the same, leading to improper segregation, making the total waste infectious. This causes a huge problem not just for the waste handlers but for the rest of the population. Inadequately channelled drainage systems are also a considerable problem facilitating the spread of resistant pathogens from healthcare settings to communities. It is not uncommon to find drainage systems from hospital facilities directly discharged to water bodies and sometimes to crop farms.

Another primary concern in terms of infrastructure is the limited diagnostic infrastructure within the country. Only about 6% of public health facilities in Nigeria have a laboratory, and two-thirds of these laboratories do not have adequate qualified personnel to handle the assigned diagnostic tasks (11). The case is not different in the animal health sector with insufficient government-owned veterinary diagnostic laboratories. Where available, these laboratories experience minimal patronage often blamed on cost and sometimes associated with low awareness on the importance of laboratory testing. From the providers' perspective, antimicrobials are prescribed based on personal judgement and at available clinical judgement rather than on susceptibility testing often because of the long wait between sample submission and completion of the laboratory testing. In the animal health sector, the insufficient government-owned veterinary diagnostic laboratories and lack of rapid diagnostic tests for use on the fields and ambulatory services also force veterinarians to use broad-spectrum antibiotics to treat several conditions (12). It is worth noting that the country is not in short supply of privately-owned laboratories, especially for human use. Where available, these laboratories experience minimal patronage often blamed on cost and sometimes associated with low awareness on the importance of laboratory testing, susceptibility tests inclusive. However, from the client's perspective, the high cost of accessing these tests compared to the cost of antimicrobials incentivises a preference for over-the-counter self-medication instead. The case is not different in the animal health sector with insufficient government and privately owned veterinary diagnostic laboratories.

The state of some healthcare facilities in the country discourages the participation and commitment of healthcare workers. Some rural areas have neither functional primary healthcare centres nor access to physicians or professionals. In some cases, physicians are only able to visit the facilities, either fortnightly or monthly. This situation is problematic for the fight against AMR as these patients and those in many Hard-to-Reach areas are deprived access to antimicrobials when they need them. This lack of access to quality antimicrobials drives them to use other antimicrobials prescribed for different purposes or those left-over by family members. In some circumstances, there is no access to antimicrobials at all, in which case the infection gets worse and further spreads within the community with a concomitant rise in morbidity and mortality.

Furthermore, only 26.5% of the Nigerian population use improved drinking water sources and sanitation facilities (13). About 47 million people in deprived areas use the open defaecation system. This practise increases the spread of pathogens—and where present the transfer of resistant genes into the environment where it comes in contact with plants, water bodies, animal and humans.

### Low Uptake of Vaccine in Some Regions of the Country

Vaccines play a critical role in preventing infections and saving lives. However, scepticisms occasioned by anti-immunisation rumours, insecurity and myths were previously noted as contributing to the low uptake of some vaccines in some parts of the country (14).

There is the myth that some vaccines are produced explicitly with developing countries in mind; with a number of them incorporated with mutagens that cause congenital disabilities and a reduction in fertility. In the wake of the increasing burden of antibiotic-resistant infections, it becomes pertinent to enhance uptake of the existing vaccines to reduce the use of antimicrobials and associated AMR (15).

The animal health sector has been able to sustain local production of veterinary vaccines against a limited number of poultry and livestock diseases by the National Veterinary Research Institute. However, the demand for vaccines far outweighs the supply. Vaccines imported largely from Asia are recruited into use without regard for their effectiveness against local strains, reducing their efficacy and overall value in disease prevention.

### Low Level of Awareness of AMR Among the Public and Professionals

Despite the potentially dire consequences of AMR on animal and public health in sub-Saharan countries, public awareness remains poor. Also, many professionals within the various One Health sectors are poorly aware of their role in ensuring antimicrobial stewardship (AMS) (11, 12, 16–18). There is still a limited appreciation of collaborative planning and data sharing needed to guarantee an enhanced response to the challenge of suboptimal antibiotic use and to mitigate the spread of AMR. Health professionals have some leverage in their relationship with clients to serve as change agents for optimal use of antibiotics, but there is still a long way ahead in ensuring that professionals adequately educate their patients or clients on AMS without seeing this as a waste of valuable time. In many cases, many patients do not understand the implication of self-medication or the importance of using antimicrobials only when it is necessary and recommended by a physician. Experiences were shared where some professionals go the extra mile of removing medicine labels before handing them over to the patient for fear of losing their patronage and ensuring that patients and clients do not purchase the same medicines without consultation or prescription. This approach is counterproductive in many ways because it neither foster transparency nor changes people's behaviour regarding AMU.

## Way Forward

Operationalizing One Health interventions in the country can significantly facilitate processes that will slow down the spread of AMR in the country. There is a pressing need for substantial investment in leadership and coordination around AMR not just at the higher level and government institutions but also across healthcare settings, research and academic institutions. Civil society groups and community members must demand greater action and strategic commitment against AMR from the political and policy leaders in the various sectors. As a problem that mirrors societal complexities, the call to actions on AMR should be hinged on locally determined evidence of the AMR burden, its impact and the potential dangers of inaction.

Considering Nigeria's social complexity, it will be helpful to deepen a bottom-up institutional stewardship by constituting Antimicrobial stewardship committees within the various sectors in line with the tiered organisation of the Nigerian health systems. In doing this, AMS roles need to be clearly defined to include linkages with other health sectors at all levels. For example, the Medical, Veterinary and Environmental Officers of Health at the district level (referred to as Local Government Areas in Nigeria) should be empowered to discharge the community health protection duties in a genuinely cooperative and interdependent manner. This becomes easy to appreciate when one admits that optimising antimicrobial use will require inclusive discussions and collaborations that empower lay population and communities and allow for their self-determination.

Efforts at repurposing the public health system for One Health should avoid duplication of efforts and aim to streamline existing responsibilities within and across sectors. For example, the Ministry of Environment needs to articulate its AMS/AMR goals and identify responsible agencies, especially regarding monitoring of contamination of the environment with antibiotic residues, standards development and risk mapping for the spread of AMR.

To strengthen the future workforce on AMR, academic institutions need to incorporate AMR's teaching from a One Health perspective into the curriculum and encourage multi-disciplinary research collaboration even before graduation. Approaches must emphasise increased reflections of emerging professionals on how we live and interact with animals, and the larger environment, as a determinant of our collective health. In particular, the training of formal prescribers and managers of antibiotics in human and animal health on the rational use of antimicrobials must be prioritised since these sectors are major hotspots for antibiotic consumption in Nigeria (11, 19). These trainings should be adaptable to identified peculiarities of the local health facilities that drive inappropriate use and aim to develop sustainable local capacity for antibiotic stewardship and improved patient care. Every hospital and healthcare centre should have clinical guidelines to inform therapy, and the authorities in charge empowered to put systems in place to check that prescribing guidelines are strictly followed. Here, the national strategy for antimicrobial consumption and resistance surveillance at the national level is needed to define and support what should be happening at the regional and local health systems levels. Prescribers must ensure that they educate their clients to understand why an antimicrobial has been prescribed and why such antimicrobials must be handled with care and taken responsibly.

A greater level of attention must be paid to low-skilled workers that clean laboratories, abattoirs, drug manufacturing companies and domestic sources, who should be enlightened on proper waste management as their routine activities may contribute to environmental contamination. Patent medicine vendors should not be left behind when designing programs on antimicrobial stewardship. They are often the closest to the rural populations, hence remains a critical stakeholder to influence behavioural change and encourage responsible use of antimicrobials. This necessitates the need for education targeted at the informal health sector workers to raise awareness and empower them to lead the change in their communities of practise.

Improving hygiene and sanitary infrastructure must be a priority for the country if we must and reduce AMR and improve public health. The opportunity provided by primary health centres (PHC) to control simple infections remains underutilised, hence the reliance on over-the-counter medications and self-medication. Strengthening PHC facilities (particularly manpower and diagnostic infrastructure) will also reduce health inequalities, including controlling simple infections, hence reducing the reliance on over the counter medications and self-medication. An opportunity exists with the Universal Health Coverage project of the Nigerian Government funded through the Basic Healthcare Provision Funds. For long term benefits, health systems strengthening initiatives must promote IPC interventions to evolve sustainably beyond the donor funding cycles.

Regulatory agencies should effectively play their roles across One Health sectors in controlling standards of practice; they must be seen to take more decisive actions regarding over the counter sale of antimicrobials for both humans and animal health. Some sector-specific actions, including strategies already developed for the regulation of veterinary pharmacovigilance aimed at establishing an effective mechanism for monitoring the use of veterinary medicinal products within Nigeria should be implemented.

At the heart of operationalising One Health is the need to mainstream AMR into other educational and public discussions that emphasise AMR management as a healthy public policy. Firstly, the country needs to rethink and reframe the way we communicate AMR especially due to poor understanding of AMR by the lay public and even among health professionals (16, 17). The challenges AMR pose—including economic losses—need to be presented as a problem of the present and the future, and communicated appropriately for adequate risk perception. The political establishments, business sector, and society are more likely to act and change behaviour if they understand the implications and the overall cost of inaction at the individual and community level. Factors driving indiscriminate use in Nigeria are intertwined in society's socio-cultural fabric that it is not conceivable to aim for behavioural change without placing the people at the centre of AMR control efforts. Therefore, equal attention should be given to people at the grassroots (farmers, consumers, patients and their relatives and other members of the community). Communication strategies should scale down AMR information in a way that is participatory, user-friendly, and respectful of local culture. Only when communities appreciate the precious nature of antimicrobials and the personal benefits of their preservation will they likely change behaviour. Like for the acceptability of many public health goods, religious and traditional leaders are an indispensable asset in mobilising community efforts and will play strong roles in the fight against AMR.

## Conclusion

A holistic strategy addressing many of the aforementioned issues will go a long way in addressing the socio-economic drivers of fear, a recurring factor contributing to inappropriate antimicrobial use among consumers and health providers in Nigeria.

With competing needs for limited resources, One Health interventions will only be sustainable by efficiently mobilising local resources, and providing a co-equal ground for disparate professionals to jointly develop and implement interventions including research that are actionable enough to inform policy and solutions for the health and economic consequences of AMR. Working in silos will undermine the AMR response but also other emerging health problem as Nigeria continues to suffer a cycle of infectious diseases that emerge as animal-human crossover outbreaks. Opportunities like this high-level interaction described in this white paper targeting an expanded One Health professionals (veterinarians, animal health care professionals and extension workers, agriculturists, environmental health workers, anthropologists) for multi-disciplinary collaboration are necessary, to drive and sustain support for One Health in public health policy discourse. Future conversations and studies should include civil society, journalists and other community-based actors to engender a whole society buy-in necessary to drive the response to an emerging complex problem like AMR.

## Data Availability Statement

The raw data supporting the conclusions of this article will be made available by the authors, without undue reservation.

## Author Contributions

The study was conceptualised by CA and OA and developed with GM. The initial draft was written by CA, OA, and GM. AE, DO, and JK critically reviewed the initial draft. All authors further reviewed and approved the final manuscript.

## Conflict of Interest

The authors declare that the research was conducted in the absence of any commercial or financial relationships that could be construed as a potential conflict of interest.
